# Experiences of Racial Microaggression Among Migrant Nurses in the United Kingdom

**DOI:** 10.1177/2333393614532618

**Published:** 2014-06-03

**Authors:** Emee Vida Estacio, Sirandou Saidy-Khan

**Affiliations:** 1 Keele University, Staffordshire, United Kingdom

**Keywords:** diaries / journals, health care, work environment, immigrants / migrants, psychology, racism

## Abstract

In this article, we explore the experiences of racial microaggression among migrant nurses in the United Kingdom. Eleven migrant nurses kept a reflective diary for 6 weeks to record and reflect on their experiences of living and working in the United Kingdom. The diary entries were then thematically analyzed. The results suggest that migrant nurses experienced racial microaggression from patients and colleagues through racial preferences and bullying. Institutional racism also hindered their opportunities for further training and promotion. As a result, some experienced feelings of anger, frustration, and even paranoia. Despite the negative consequences of racial microaggression on their emotional well-being, incidents were downplayed as trivial because of their vague and subtle nature. To encourage better multicultural interactions in the workplace, supportive organizational infrastructures need to be in place to enhance diversity awareness and to improve mechanisms for reporting and dealing with cases of racial microaggression.

Racism is a continuing problem within the health care sector in Europe (Bernardotti, Dhaliwal, & Perocco, 2007). Despite existing legislation against racial discrimination within the United Kingdom, evidence of racism in the National Health Service (NHS) is widely documented (Mistry & Latoo, 2009). In 2003, the former chair of the Commission for Racial Equality, Sir Trevor Phillips, criticized the NHS for institutional racism and highlighted the prevailing issue of its “snowy peaks,” that is, all White at the top (Duffin & Parish, 2005). The Parekh Report (2008) also noted how the vast majority of employees from Black and minority groups in the NHS were found in lower grades, and were underrepresented at senior levels. In fact, only 1% of chief executives within the NHS were from Black or minority ethnic groups (Carvel & Gentleman, 2009).

There are growing discussions concerning what are being labeled as “new forms of racism.” In contrast to “old forms,” which were described as overt, blunt, and hostile, contemporary forms of racism are considered covert, subtle, and sometimes unintentional (Dovidio, Gaertner, Kawakami, & Hodson, 2002). Contemporary forms of racism are difficult to challenge because quite often its perpetrators are not aware that their actions can be considered racist. New forms of racism can be problematic because these reflect deep-seated prejudices that can be difficult to overcome.

Racism stems from historical relationships between groups of people wherein power is asserted unjustly to establish one group’s dominance over another (Durrheim, Hook, & Riggs, 2009). Racial hierarchies can be produced and preserved over time through racist attitudes, discourses, and activities. Individuals can thus behave in a racist manner without the intention to cause racial offense to others; rather, such behaviors are performed simply to protect advantaged racial positions within the wider social hierarchy.

Discursive analyses of prejudiced talk also suggest that such behaviors could result from micro-level dynamics within a social interaction. Racially prejudiced talk could be used for purposes other than to express negative attitudes or to defend social positions. For example, it can also be used for other communicative social functions such as to amuse, to attract or divert attention, to express intimacy or solidarity, or to lighten up the conversation (Condor, 2006). The trouble with these new forms of racism, however, is that some behaviors can have the opposite effect than what was intended.

It has been suggested that racism continues to persist in modern society because it has a basis in people’s everyday lived experiences (Durrheim & Dixon, 2005). Although it is important to explore the broader social and historical context of racism, it is equally important to consider the embodied materiality of everyday life and how racism takes its form at this level (Hook & Howarth, 2005). Thus, as Durrheim and Dixon (2005) suggested, “[t]he social psychology of racism needs to take seriously people’s lived experiences of race relations. This involves an empirical focus on social life in ordinary contexts in which everyday practices are structured around ‘race’” (p. 446). Under the umbrella of contemporary racism, Sue, Capodilupo, and colleagues (2007)reintroduced the term “racial microaggressions,” which are defined as “brief and commonplace daily verbal, behavioral, or environmental indignities, whether intentional or unintentional, that communicate hostile, derogatory, or negative racial slights and insults” (p. 271).

Racial microaggression can be classified into three forms (Sue, Capodilupo, et al., 2007): (a) *microassaults*, which are explicit expressions of racial derogation, (b) *microinsults*, which are behavioral or verbal acts that express negative attitudes toward another person’s racial identity or heritage, and (c) *microinvalidations*, which are actions that invalidate another person’s thoughts, feelings, and experiences in relation to racial or ethnic identity. Examples of racial microaggression include treating someone as a foreigner in his or her own land (e.g., complimenting an Asian American for speaking fluently in English), treating someone as a second-class citizen (e.g., refusing or delaying services to people from Black or minority ethnic groups), or denying one’s subjective perceptions of racism (e.g., telling someone not to be too sensitive over racial issues).

It is possible that well-intentioned individuals could commit racial microaggressions without their knowledge because these can occur outside their conscious awareness. As a result, individuals could engage in racially prejudiced behavior without feeling guilty about it (Sue, 2003). Nonetheless, racism, whether it is deliberate or not, negatively affects the well-being of its targets (Deitch et al., 2003; Jasinskaja-Lahti, Liebkind, & Perhoniemi, 2006). Everyday experiences of racial discrimination have been associated with increased incidence of chronic illnesses (Gee, Spencer, Chen, & Takeuchi, 2007) and mental health problems (Borrell, Kiefe, Williams, Diez-Roux, & Gordon-Larsen, 2006). Because of the subtle nature of racial microaggressions, targets also often face the additional emotional burden of not overreacting (Wang, Leu, & Shoda, 2011). As a consequence, some are forced to downplay an incident by dismissing it as a “joke” (Burdsey, 2011). Some are also left to question whether an incident was actually racist or not (Sue, Capodilupo, et al., 2007). At worse, racism can be internalized, which helps promote the justification of racially prejudiced activities (Larsen, 2007).

One way to address the negative consequences of racial microaggression is to make the “invisible” visible (Sue, 2004; Sue et al., 2008). Understanding patterns of racial microaggression can help explore the nature and impact of these incidents. In doing so, plausible mechanisms to overcome them can also be explored. In this article, we aim to contribute to this area of research by exploring the experiences of racial microaggression among migrant nurses in the United Kingdom. Our research builds on previous findings that suggest that migrant nurses working in the NHS have experienced racial discrimination from both patients and colleagues (e.g., Ball & Pike, 2009). Although survey-based studies have been conducted to explore this issue, the use of qualitative methods can add insight into the social psychology of racism by placing fewer constraints on what constitutes racism (Durrheim & Dixon, 2004). Thus, in the current study we used a qualitative, diary-based approach as part of a wider study on life as a migrant nurse in the United Kingdom.

## Method

Diarywriting is a data collection method wherein participants keep a diary of experiences or events related to the topic of interest over an extended period of time (Wilson & MacLean, 2011). This method is quite flexible and can be used both quantitatively and qualitatively. For example, diaries have been used to quantify alcohol consumption and examine its relationship with implicit self-esteem and interpersonal interactions (DeHart, Tennen, Armeli, Todd, & Mohr, 2009). Diary studies have also been used to examine the effect of anger and anger regulation styles on everyday pain experienced by women with fibromyalgia (Van Middendorp et al., 2010).

Qualitatively, diaries can be used as a reflective tool to enable participants to record and reflect on their thoughts, feelings, and experiences of a particular phenomenon. For example, diaries have been used to explore how cancer surgery patients cope with alterations to their appearance after facial surgery (Furness & Garrud, 2010). Diaries have also been used as an empowerment tool to enable patients to record and communicate their experiences of their illness and to explore meaningmaking as part of the process (Stensland & Malterud, 1999).

Diaries can be a useful way to generate in-depth information about people’s experiences based on their point of view. Diaries can provide participants with the opportunity to steer the direction of the research by highlighting issues that are most important to them. Diaries have been particularly effective in eliciting sensitive and personal reflections that might otherwise be undisclosed through interviews or focus group discussions (Day & Thatcher, 2009). Furthermore, diarywriting can also have a therapeutic effect by enabling participants to share their experiences and to reflect on these within a confidential environment (Wright, 2009). Thus, diarywriting could be an effective way to explore everyday racial microaggressions because this method can provide participants with a safe and personal space where they can express their views and experiences.

### Participants

Twelve women migrant nurses from two hospitals in the midlands of England agreed to take part in this study; 9 were originally from the Philippines, 1 from Kenya, 1 from Zimbabwe, and 1 from Zambia. The duration of their stay in the United Kingdom ranged from 6 to 11 years. Participants were recruited through hospital-based email lists, posters, and word of mouth. Eleven completed the diary-writing process and 1 (from the Philippines) dropped out for undisclosed reasons.

### Researchers

It is vital that researchers’ values and assumptions are made explicit and reflected on when conducting qualitative research. For this study, our team comprised two researchers who were originally from the Philippines (Estacio) and Gambia (Saidy-Khan). We also received mentorship and research advice from a senior colleague. We both have an interest in race equality, particularly in health care provision and access. We both have also experienced racial microaggression at some point during our stay in the United Kingdom. We are passionate about issues relating to racial prejudice and inequality. Estacio, in particular, had been involved in organizing staged protests against racism. We discussed potential sources of bias before and after data collection and analysis. We also consulted theparticipants after the data analysis to ensure that we had interpreted their responses appropriately.

### Procedure

After gaining NHS ethics and hospital research and development approval, migrant nurses from the partner hospitals were invited to a project initiation meeting. The aim of this meeting was to discuss the research with potential participants before they provided their consent to participate. Those who agreed to take part were asked to keep a reflective diary over a period of 6 weeks. They were asked to record their experiences and reflections on their life as a migrant nurse in the United Kingdom. The participants were not obliged to write on a daily basis; instead, they were encouraged to write whenever they felt like writing.

The purpose of the diarywriting was to provide them with a space to share their thoughts and stories with us. Although participants were given some guidance on what to write in their diaries (e.g., motivations for coming to and staying in the United Kingdom, pre-arrival expectations, what makes a day good or bad, costs and benefits of being in the United Kingdom, plans for the future, any other issues), they were encouraged to write whatever they wanted to share. It is worth noting, however, that although this article is about their experiences of racial microaggression, we did not prompt theparticipants to write about this.

During the 6-week period of diarywriting, participants were contacted byphone by a member of the research team every 2 weeks to monitor progress and to offer support if needed. Amazon gift vouchers worth £100 each were also offered to compensate for their time and contribution. At the end of the writing period, a World Café event was organized to bring all of the participants together. A World Café is a facilitated discussion wherein participants are encouraged to share their views with others in a comfortable and relaxed environment (Brown & Isaacs, 2005).

During this event, the participants submitted their anonymized diaries. They were also given the opportunity to share with the other participants their experiences of diarywriting. They also discussed some of the themes that came out from their diaries. This event aided data analysis by allowing the participants to highlight and elaborate on the key points expressed in their individual diary.

After this event, we transcribed the diaries verbatim, and the transcripts were then analyzed thematically, following the procedures suggested by Braun and Clarke (2006). In this case, we initially read the diaries independently. Each of uswrote down notes and produced codes from the text. Both of us then collated the codes to produce preliminary themes. The themes were then reviewed and checked in relation to the original text and codes. After rigorous discussion and cross-checking of codes and raw data, several themes emerged, which included the participants’ early experiences in the United Kingdom, management and NHS-specific issues, language barriers, work–life balance, and racial microaggression.

A stakeholder event was organized toward the end of the project. Participating nurses and their families were invited, as well as other hospital staff, the media, and other interested parties. Posters were also prepared to summarize themes from the diary extracts. The participants were given the opportunity to review the content of the posters and to comment on these before printing. They also gave their consent to make the posters available for public viewing.

## Analysis

The participants’ diary entries reflected incidences of racial microaggression. The scope of these incidents ranged from racial preferences from patients to racist bullying from colleagues. Institutional racism was also discussed, andhow the participants learned to accept these incidents as part of their everyday working environment.

### Patients’ Racial Preferences

It is not uncommon for patients to express their preferences when they are taken into care. Diary entries from this study reflected how some patients showed racial microaggression by turning down services offered by foreign nurses; for example,

A male patient who was transferred asked to be taken to toilet. Every time I answer for his buzzer, he will say he doesn’t need the toilet. Another foreign nurse answered his buzzer and the same answer. When an English staff answered his buzzer he gladly went with her. It was only a suspicion that he didn’t like foreign nurses but it came to light when all his family came and asked for an English nurse to take him to the toilet.

Showing preference for a nonmigrant nurse more than an equally capable foreign nurse is a form of racial microaggression because it gives the impression that the patient considers one group of nurses to be more competent than another. Participants also mentioned that some patients preferred British nurses because they assumed that migrant nurses were unable to speak English, despite the fact that fluency in English is a requirement for foreign nurses working in the NHS. One participant wrote, “The family was asking for an English nurse to update them with their patient’s medical information although I know I can speak English clearly and fluently.”

The Nursing and Midwifery Council in the United Kingdom sets very high standards of English-language proficiency for migrant nurses. Applicants from outside the European Economic Area are required to take the academic version of the International English Language Testing System and to achieve an overall average score of 7 (out of a possible 9), with at least a score of 7 for each section (i.e., listening, reading, writing, and speaking). Achieving this score means that the applicant can use the language at an operational level and should be able to handle complex language well. This level of English proficiency is sufficient to enable foreign nurses to communicate clearly and effectively with colleagues, patients, and patientcarers.

The participants expressed the view that they always had their patients’ best interests at heart. Although racial preferences amongpatients exist, participants in this study had learned not to holdthese against them. Rather than taking racial microaggression personally, participants looked on these incidents within the historical and social background of their patients: “Sometimes it seems like some service users are not thrilled by my skin color but I look at their age and think they have lived through times when racial discrimination was not frowned upon.”

Considering the wider context, and locating patients within it, allows migrant nurses to maintain a good working relationship with their patients, despite incidence of racial preference. This enables them to carry out their duties as nurses without feeling resentful when experiencing racial microaggression from patients.

### Racist Bullying From Colleagues

The participants also shared their experiences of racist bullying from some of their colleagues. Although there were occasions when they felt welcomed and accepted, there were instances when they felt segregated from the other nurses:

The staffs were good but I found the staff nurses on the same level were quite rude, proud to work on same level with me. We are three, they stick the two together and gossip and laugh behind your back. If you asked them something they wouldn’t help.

Unlike the “old forms” of racism, new forms of racist actions are more subtle. Rather than using explicit and derogatory actions, new forms of racism use exclusion to make their targets feel unwanted and isolated from the group. At times, the use of humor can also achieve this outcome; for example, one participant wrote, “I can’t remember what it was all about but what I can remember, she called me a cheeky monkey and I wasn’t happy with the language and it made me miserable and tearful.”

Some people might not consider the use of the term *cheeky monkey* as racist; however, context should always be taken into consideration when dealing with this issue on racial grounds. Although “cheeky monkey” can be used as a playful term of endearment, it can also be used to attack someone racially through tone, manner, and emphasis on certain words. These subtleties make this form of racist bullying more difficult to challenge. Once taken out of context, the prejudiced intentions of these encounters might not be as easy to prove.

Although racial microaggression can be achieved through subtle means, theparticipants also shared their experiences of more blatant forms of racist bullying. There were instances wherein hurtful words and actions were taken to demean migrant nurses and to make them feel less competent:

A patient was struggling to evacuate her stool, being hoisted for aid transfer; she was half “hanging.” The assigned nurse is bathing another patient. For the patient’s best interest, I went to help her evacuate manually/digitally. She was relieved and thanked me after a successful bowel movement. Then her nurse came out and I went to tell her. She went like a bottle of pop and told me that this is an incident report and that I am not qualified to do it. . . . Well, I’m a graduate of Bachelor of Science in Nursing. I have a degree . . . Fortunately, the doctor at that time backed my good work to the patient. But still the nurse who was only in the program for three to four years wrote an incident report and told me I am only a pebble and she is the rock.

Based on the participants’ diary extracts, it is not uncommon for migrant nurses to experience this level of antagonism from other nurses. There were frequent accounts oftheir skills and qualifications being downgraded by others. Surprisingly, it is often nurses within the same pay grade or lower (e.g., health care assistants) who express negative attitudes toward them: “As nursing qualified, I ended up doing health care assistant job wherein at the same time health care work assistants are empowered to toss you ‘round then frustration builds up eventually.”

It could be said that negative behavior against migrant nurses might stem from internal competition for promotion between nurses. There were also wider discourses within the local area wherein migrant workers generallywere blamed for “stealing” local jobs. These factors can contribute to the frustration experienced by both domestic and migrant nurses, which can further aggravate the stressful working environment that nurses are already exposed to.

### Institutional Racism

The NHS has generally been criticized for institutional racism. Nursing practice is no exception. Based on the participants’ diary extracts, racial biases exist in nursing practice, particularly in terms of recruitment and progression. One participant wrote, “This manager seemed to be a bully, employed white nurses and make them in charge. At least I was the longest service there and I felt racism amongst the workplace.”

The participants also expressed discontent concerning the seemingly unbalanced allocation of training courses. There was a common theme in this study ofsome migrant nurses being denied opportunities to develop their knowledge and skills:

I have also questioned the criteria of sending nurses for different courses within the Trust. I always put my name first on the list but to my amazement I have never been listed for interviews. Nurses who actually joined later than me or who were students go for the courses. I remember one simple one-day course where the list was put when I was on shift and I put my name down. My name never appeared on the list. . . . I had to contact the one who was in charge and told me my name was not on the list. I was so demoralized that I thought, I would never put my name on any list. I feel when I go back home I will be still the same nurse I came with the same qualifications. I expected that when I go back home I will be holding degrees and different courses of achievement but I feel my nursing colleagues back home will be far better than me in education.

Perhaps there is a misconception that migrant nurses come to work in the United Kingdom for financial reasons only. However, as reflected in our findings, migrant nurses who come to work in the United Kingdom also hope to gain new knowledge and skills during their stay. For some, work in the United Kingdom is only a springboard for their ambition to work in other countries (e.g., Australia, the United States, New Zealand). Withholding opportunities for further training from migrant nurses could prevent these nurses from improving their skills and could also stunt their prospects for career advancement, either in the United Kingdom or elsewhere. Because of the lack of further training, it is not uncommon for migrant nurses to see their juniors overtake them in their careers. One participant wrote,“I have seen final year students, mentor them and only to see them being my seniors. I have learned to accept this as long as I am doing my work and demonstrating my good nursing standards.”

### Accepting Racial Microaggression

Experiencing racial microaggression in the workplace can have adverse consequences on employees’ emotional well-being. Some of theparticipants disclosed feelings of inferiority as a result of working with racially abusive colleagues:

I have always felt different and inferior. I think this comes from being black and always feeling that others are better. Even now sometimes at work I feel inferior. Mainly it depends on who is at work with me. Some people have a way of making me feel very small and others have a way of making me feel equal to them.

The subtle nature of racial microaggression can also make its targets feel somewhat confused about the experience itself. On some occasions, because of its vagueness, nurses were unable to distinguish whether they were experiencing racial microaggression or not. To err on the side of caution, some participants would rather admit to being paranoid than to suspect their colleagues of being racially aggressive:

Sometimes I think I am getting paranoid. Like last night, I felt a bit overwhelmed by the amount of work I had to do each time I saw two colleagues talking together I had a feeling they were commenting or discussing my inadequacies. Soon after that they would be good to me or just usual and I did not know what to make of it. Since I could not hear what they were saying and there was no other indication that they were talking about me. I put it down to paranoia.

When it comes to blatant forms of racism, individuals react differently depending on factors such as the gravity of the incident, the aggressors’ and targets’ personalities, and their perceived support for action. When targets are not empowered to voice their concerns, it is unlikely that they will be able to protect themselves from these encounters or to take appropriate action. There might also be others who simply ignore what happens, who work as they normally do or pretend that they are “against a brick wall.” Although some participants confronted racial microaggression directly by reporting these incidents to their managers, there were instances when their grievances were "swept under the rug" to prevent issues from escalating further:

One day, one of the health care assistants worked with me and started with the same attitude of intimidation, bullying etc. As I have been told from other foreign nurses who have worked with her, I did not want this to continue as usual, I had to tell that, “everyone has been complaining and we feel you are a racist or bullying us.” From there she went to the sister who at the same time was the same like others. The sister told her to report to the matron, without even calling us to hear what happened. Anyway I explained to the matron what went wrong and about other complaints from other foreign nurses. . . . She said she talked to her and let us just close the problem.

Downplaying racial microaggression encourages employees, both domestic and migrant, to simply accept itas part of their everyday working life. Perhaps it is more convenient to ignore racial microaggression to enable nurses to deal with more pressing and life-threatening situations that confront them on a daily basis; however, accepting racial microaggression as part of the daily working environment only masks the issue, if not denying that it exists entirely.

## Discussion

In this study we aimed to explore the experiences of racial microaggression among migrant nurses in the United Kingdom using a diary-based approach. As Durrheim and Dixon (2005) suggested, racism continues to persist in modern-day society because it has a basis in people’s daily experiences. It is important to explore racism at the micro level to enable us to understand how it is embodied in everyday life (Hook & Howarth, 2005). The results from this research support previous findings suggesting that racism exists within the NHS (Mistry & Latoo, 2009). At the micro level, migrant nurses experienced racial microaggression from both patients and colleagues through racial preferences and bullying. Institutional racism also hindered these nurses’ opportunities for further training and promotion. As a result, some experienced feelings of anger, frustration, and even paranoia. Despite the negative consequences of such racial microaggression on their emotional well-being, these incidents were downplayed as trivial because of their vague and subtle nature.

The use of diaries to generate insights from participants proved to be an effective strategy for this study. As discussed earlier, racial microaggression was not the original focus of this project. Our interest in exploring and writing about this issue resulted from the participants’ disclosures through their diaries that racial microaggression was one of the key issues that they confronted as migrant nurses in the United Kingdom. By providing them with a safe and confidential space to share their stories, diarywriting enabled them to steer the focus of the study to the matters that were most important to them.

As a limitation, it is perhaps worth noting that the participants had been in the United Kingdom for a considerable amount of time (i.e., 6 to 11 years). This could mean that they might have learned how to cope with racial microaggression, considering that they continued to live and work as nurses in this country. It is also possible that the level of racial microaggression experienced by theparticipants could be less severe than that experienced by those who had either voluntarily or forcibly left their post. Future research could examine the experiences of newer migrant nurses and those who have left their nursing positions in the United Kingdom.

Furthermore, although the sample size for this research was adequate, there is still scope to increase participant numbers and to widen its reach. Although generalizing from our findings was not our priority, recruiting participants from only two institutions in one region of the United Kingdom is a limitation of the study. In addition, although we attempted to recruit as diverse a sample as possible, a majority of participants were originally from the Philippines. We need to consider cultural and historical backgrounds and how these can influence the nature and extent of racial microaggression. For example, it would be worth examining how racial microaggression is experienced by first-, second-, or even third-generation migrants from previous colonies and by those who come from community groups that are currently in conflict with the United Kingdom.

In practice, organizational infrastructures need to be in place to encourage better multicultural interactions in the workplace. When cases of racial microaggression are reported, it is important that incidents are investigated and dealt with accordingly. Passive acceptance of injustice could arise when abuse is swept under the rug. Furthermore, diversity awareness and training could be set up to help staff members be aware of the subtleties of racial microaggression. Sue (2004) was correct in his assertion that one way to tackle racial microaggression is to make the “invisible” visible. Because well-intentioned individuals might not be aware that their actions can be considered racially aggressive, discussing what is and is not acceptable could help establish mutual understanding from individuals from different racial and cultural backgrounds. This could help prevent unintentional racial microaggression from happening in the first place.
